# The Impact of Unilateral or Bilateral Cataract Surgery on Visual Acuity and Life Quality of Elderly Patients

**DOI:** 10.1155/2015/509049

**Published:** 2015-03-22

**Authors:** Lei Zuo, Haidong Zou, Xinfeng Fei, Weiqi Xu, Jianhong Zhang

**Affiliations:** ^1^Department of Ophthalmology, Branch of Shanghai Jiaotong University Affiliated First People's Hospital, Shanghai 200081, China; ^2^Department of Ophthalmology, Shanghai Jiaotong University Affiliated First People's Hospital, Shanghai 200080, China

## Abstract

In the current study, the CLVQOL was used to assess VRQOL before unilateral or bilateral cataract surgery and at the end of the follow-up period in order to determine the greater beneficial mode of surgery for patients, if one of the two surgical methods is more beneficial over the other. The patients were classified as receiving unilateral (group A) and bilateral cataract surgery (group B). There were no significant differences between groups A and B before the operation in terms of life quality scores, binocular weighted average LogMAR BCVA, age, educational level, gender, systematic and ocular comorbidities, and the complications of the operation. It was shown that visual acuity improved more significantly with bilateral cataract surgery than with unilateral surgery in elderly patients with a high preoperative disease burden in Shanghai city. However, the improvement in life quality was not different in patients receiving either bilateral or unilateral cataract surgery.

## 1. Introduction

Cataract is one of the common causes of visual impairment in people over 40 years old, which has a negative impact on their quality of life. Both unilateral and bilateral cataract surgeries have been shown to effectively improve the visual function of cataract patients. Therefore, it is important to elucidate the impact of unilateral and bilateral cataract surgeries on the improvement of visual acuity and quality of life of these patients and to determine whether bilateral cataract surgery has greater benefit over unilateral cataract surgery.

Visual acuity is commonly used to quantitatively measure the visual function of patients in clinical practice; however, it is the patient's self-perceived visual function and improvement in the life quality that are gradually recognized by the clinical practitioners and used as important factors to evaluate the outcomes of cataract surgery. The scale developed by Mangione et al. [1–3] has the advantage of effectively evaluating the impact of visual function on daily life and the alteration of life quality by the treatment. In the current study, the Chinese-version low vision quality-of-life questionnaire (CLVQOL) [4] was used to assess vision health-related quality of life (VRQOL) [5] before unilateral or bilateral cataract surgery and at the end of the follow-up period [6] in order to identify potential impact factors on the surgery outcome and to determine the greater beneficial mode of surgery for patients, if one of the two surgical methods is more beneficial over the other.

## 2. Methods

### 2.1. Patients

A total of 335 cataract patients underwent phacoemulsification surgery at the branch of Shanghai Jiao Tong University Affiliated First People's Hospital between January 2012 and February 2013, among which 37 patients were lost to follow-up. As a result, the complete follow-up records and the corresponding questionnaires were obtained from 298 patients. There were 165 female and 133 male patients enrolled in the study, with the average age of 73.53 ± 10.09 years (range, 43–96 years). The patients undergoing unilateral cataract surgeries were classified into group A (153 eyes from 153 patients), and those undergoing bilateral cataract surgeries were classified into group B (290 eyes from 145 patients). The CLVQOLs questionnaires were completed by each patient before surgery and 3 months after surgery, and 30 patients received an additional questionnaire inquiry using the identical scale 2 weeks before the operation.

### 2.2. Cataract Surgery

Cataract surgery was defined as the absence of the crystalline lens in at least one eye of the subject [7], who received the phacoemulsification in combination with foldable intraocular lens (IOL) implantation. The IOL was foldable with single focus, and the patient paid 30% of the IOL cost. Bilateral cataract surgery was performed sequentially. Time interval was 2 to 3 weeks.

### 2.3. Assessment and Examinations

The demographic information of the patients was collected including gender, age, and educational level. The detailed ophthalmological examinations included the visual acuity test, anterior eye segment examination, and fundus examination before operation and 3 months after operation. At the same time, each patient was also required to complete the CLVQOL. The visual acuity was measured using an international standardized visual acuity chart. The best corrected visual acuity (BCVA) (subjective optometry) was converted into the minimum angle of resolution (LogMAR) vision. When Snellen visual acuity was less than 0.01, the visual acuity of “hand-motion” perception was defined as LogMAR2.2, that of “counting fingers” as LogMAR2.3, and that of “light perception” as LogMAR2.5 [8, 9].

### 2.4. The Assessment of Vision Health-Related Quality of Life

The CLVQOL questionnaire used in the current study was originally acquired from the low vision health-related quality-of-life questionnaire (LVQOL), which was developed by Wolffsohn and Cochrane [5], and translated into a Chinese version that was modified and culturally adapted for the Chinese patients [4]. The scale consists of 25 items, including four scales, namely, the Distance Vision (reading road signs or watching TV), Mobility and Lighting (outdoor activities and crossing a street with traffic), Adjustment (expectations on quality of life and perceived visual acuity), and Reading and Fine Work and Activities of Daily Living (reading the clock, reading one's own handwriting, and daily activities). Each item was scored using a numeric scale ranging from 0 (worst) to 5 (best). The total highest score was 125, and the score was correlated with the level of quality of life; the higher the score, the better the quality of life. The questionnaires were completed through face-to-face patient interviews conducted by two well-trained investigators. The VRQOL scores were calculated, and any complications due to cataract surgery [10] were also recorded for subsequent analysis.

### 2.5. Statistical Analysis

The software Statistical Package for the Social Science, version 11 (SPSS 11.0), was used in the current study for statistical analysis. *P* < 0.05 was considered statistically significant. The Spearman rank correlation method was used to analyze the correlation between the binocular weighted average LogMAR BCVA (with the weight of better eye and worse eye taken as 0.75 and 0.25, resp. [11]) and the VRQOL before surgery and at the end of follow-up. The correlation coefficient between the weighted average LogMAR BCVA and VRQOL of the patient before surgery and at the end of follow-up and that between the changed values of these two variables were calculated.

The score of VRQOL and the weighted average LogMAR BCVA were compared between the two groups by using Mann-Whitney test. *t*-test and Pearson *X* ^2^ test were used for the comparison of continuous data and categorical data, with respect to age, gender, educational level, systematic and ocular comorbidities, and surgery complications. Mann-Whitney test was used to investigate the impact of systematic and ocular comorbidities, gender, and surgery complications on the alteration of VRQOL scores at the end of the follow-up period.

The independent impact factors were identified by multiple linear regressions, with the improvement of VRQOL scores at the end of the follow-up period as an induced variable, and with the weighted average LogMAR BCVA before operation, VRQOL scores obtained before operation, the alteration of weighted average LogMAR BCVA after operation, age, and educational level as independent variables. The results were presented as standardized coefficients/*P*. The classified variables for the method of operation (1 for unilateral, 2 for bilateral), surgery complications (1 for yes, 0 for no), ocular comorbidities (1 for yes, 0 for no), and systematic complications (1 for yes, 0 for no) were recorded, and their influence on the alteration of the VRQOL score (1 for a score increase of no more than 19 and 2 for a score increase of more than 20) was analyzed by logistic regression. The results were presented as coefficients/*P*.

The reliability of the study was tested by Cronbach's*α* coefficient, and the repeatability of the study was tested by intraclass correlation coefficient (ICC) [12].

## 3. Results

### 3.1. Demographic Analysis

Table 1 compares the clinical characteristics of patients who underwent either unilateral or bilateral cataract extraction. There was no significant difference between the two groups with respect to age, educational level, gender, ocular comorbidities, systematic comorbidities, and surgery complications (*P* > 0.05). The detailed information of the ocular and systematic comorbidities and the surgery complications is listed in Table 2.

### 3.2. Visual Function and Quality of Life

The binocular weighted average LogMAR BCVA was significantly increased at the end of the follow-up period in group B compared with that in group A, although there was no significant improvement in the life quality between the two groups (Table 3).

### 3.3. Association between Visual Acuity and Quality of Life

The Spearman correlation coefficients between the preoperative weighted average LogMAR BCVA and the VRQOL score were 0.792 (*P* = 0.000), 0.700 (*P* = 0.000), and 0.806 (*P* = 0.000) for patients who underwent the unilateral cataract surgery, for patients who underwent bilateral cataract surgery, and for total patients, respectively. The correlation coefficients between the weighted average LogMAR BCVA at the end of follow-up and the VRQOL scores were 0.664 (*P* = 0.000), 0.443 (*P* = 0.014), and 0.570 (*P* = 0.000) for patients who underwent unilateral cataract surgery, for patients who underwent bilateral cataract surgery, and for total patients, respectively.

The correlation coefficients between the postoperative improvement in the weighted average LogMAR BCVA and the improvement in the VRQOL score were 0.598 (*P* = 0.000), 0.707 (*P* = 0.000), and 0.739 (*P* = 0.000) for patients who underwent unilateral cataract surgery, for patients who underwent bilateral cataract surgery, and for total patients, respectively.

### 3.4. The Impact Factors for the VRQOL Score

Surgery complications and ocular comorbidities were found to be negative impact factors in the improvement of life quality, whereas gender and systematic comorbidities had little impact on the improvement of life quality (Table 4).

The preoperative VRQOL score, the increase in the postoperative weighted average LogMAR BCVA, the preoperative weighted average LogMAR BCVA, ocular comorbidities, and systematic comorbidities were the important factors that were found to have a significant influence on improving the life quality of patients. The method used for cataract surgery (unilateral or bilateral) was also identified as an impact factor, although with less significance (Table 5).

The internal consistency of responses before and after surgery, measured by the Cronbach *α*, was 0.95 and 0.92, respectively. The intraclass correlation coefficient was 0.93.

## 4. Discussion

The current study focused on the impact of unilateral or bilateral cataract surgery on vision-related life quality of elderly patients in China, with the objective to investigate factors influencing the improvement in the postoperative quality of life.

It was previously reported that the therapeutic effect of bilateral cataract surgery is better than that of unilateral cataract surgery [13]; however, it was also demonstrated that cataract surgery on the fellow eye had no obvious clinical benefit in elderly female patients [14]. In the current study, we found that both unilateral and bilateral cataract surgeries can significantly improve both visual acuity and quality of life. Successful cataract surgery may not always lead to a significantly improved life quality of elderly patients, who usually have various comorbidities and relatively lower level of education [9, 15, 16].

We showed that cataract surgery complications significantly influenced postoperative quality of life. Our findings suggest that if preoperative examinations reveal a potential risk of complications [10, 17], such as hypermature cataract [18], microcoria, serious adhesions in the posterior of the iris, posterior scleral staphyloma, and/or corneal refractive operation history that cause difficulties in the prediction of IOL diopter [19], surgeons should make sufficient preoperative preparations to avoid surgical injury. Sufficient preoperative communication with the patients would be helpful to lower their expectations about surgery outcome [20, 21], which will positively affect postoperative life quality.

In the present study, there was no significant difference in the improvement of life quality in patients with or without systematic comorbidities. In general, a comprehensive preoperative evaluation should be carried out, which includes obtaining a complete medical history and performing routine examinations. Unilateral cataract surgery should be usually preferred for those patients who have a poor general condition and are in a feared state of mind. The surgery should be based on sufficient communication between the clinicians and patients. Proper preoperative care [22] may pose lower risk of poor outcomes in elderly patients with systemic complications.

Based on the findings of the current study, cataract surgery on the fellow eye is associated with the type of ocular comorbidity being in the cataract patient [17, 23]. Primary angle-closure glaucoma is the major type of glaucoma in China [24], in which both of the eyes have similar anatomical pathology. Cataract surgery can improve the anatomical structure of the chamber angle [25]; therefore, bilateral cataract surgery is usually chosen based on the recommendation of the surgeon. The patients with diabetic retinopathy generally preferred bilateral cataract surgery. Diabetic retinopathy often affects both eyes of the patient, and bilateral cataract surgery is convenient for fundus inspection and laser photocoagulation treatment. Unilateral cataract surgery is often operated for patients who have ever undergone a retinal reattachment operation or vitrectomy. Because there is an increased need to adjust anisometropia in patients with pathological myopia and with a history of vitreoretinal operations, there is a corresponding increase in the number of patients undergoing bilateral cataract surgery.

Previous study [26] showed that the clinical outcomes and patient-rated satisfaction were similar whether bilateral cataract surgery was performed simultaneously or sequentially. In China, surgeons did not reach a consensus on option of simultaneous bilateral cataract surgery. We performed sequential bilateral cataract surgery in this study. With the development of cataract surgery techniques, there would be more doctors recognizing that simultaneous bilateral cataract surgery is safe and more economical than sequential [27], and it is very effective way to cut down waiting times in case there are long queues to surgery.

In summary, a greater improvement in visual acuity was observed in elderly patients of Shanghai city with a high preoperative disease burden and receiving bilateral cataract surgery, compared with those who received unilateral cataract surgery. However, there was no significant difference between the two groups in terms of improvement in vision-related quality of life.

## Figures and Tables

**Table 1:** Clinical characteristics compared in unilateral and bilateral cataract extraction patients.

	Unilateral cataract extraction patients	Bilateral cataract extraction patients	*P* value	Statistical methods
Age (SD) (year)	74.38 (10.82)	72.57 (9.29)	0.477	Independent samples *t*-test
Educational time (SD) (year)	7.88 (3.80)	8.80 (3.60)	0.328	Independent samples *t*-test
Gender (male, female)	66, 87	67, 78	0.594	Pearson chi-square test
Ocular comorbidities (%)	84 (54.9)	80 (55.2)	0.963	Pearson chi-square test
Systemic comorbidities (%)	84 (54.9)	85 (58.6)	0.517	Pearson chi-square test
Surgical complications (%)	10 (6.5)	9 (6.2)	0.907	Pearson chi-square test

**Table 2:** Ocular comorbidities, systemic comorbidities, and surgical complications in patients receiving either unilateral or bilateral cataract extraction, ^*^ *P* < 0.05 and ^**^ *0.05* < *P* < 0.10.

	Number (%)		*χ* ^2^/*P* (Pearson chi-square test)
	Unilateral cataract extraction patients	Bilateral cataract extraction patients	
Ocular comorbidity			
Age-related macular degeneration	11 (7.2%)	12 (8.3%)	0.136/0.712
Pathologic myopia	20 (13.1%)	16 (11.0%)	0.291/0.590
Glaucoma	21 (13.7%)	31 (21.4%)	2.836/0.092^**^
Surgery history of scleral buckling and/or vitrectomy	13 (8.5%)	3 (2.1%)	6.054/0.014^*^
Pathologic myopia and surgery history of scleral buckling and/or vitrectomy	11 (7.2%)	10 (6.9%)	0.010/0.921
Diabetic retinopathy	2 (1.3%)	7 (4.8%)	3.150/0.076^**^
Other	6 (3.9%)	1 (0.7%)	3.390/0.066^**^

Systemic comorbidity			
Hypertension	37 (24.2%)	37 (25.5%)	0.071/0.790
Diabetes mellitus	5 (3.3%)	11 (7.6%)	2.732/0.098^**^
Hypertension and diabetes mellitus	22 (14.4%)	18 (12.4%)	0.247/0.619
Cardiopathy	8 (5.2%)	11 (7.6%)	0.693/0.405
Respiratory	5 (3.3%)	3 (2.1%)	0.410/0.522
Other	7 (4.6%)	1 (0.7%)	4.302/0.038^*^

Surgical complication			
Vitreous loss	5 (3.3%)	4 (2.8%)	0.066/0.797
Iris atrophy	3 (2.0%)	3 (2.1%)	0.004/0.947
Anisometropia	1 (0.7%)	2 (1.4%)	0.393/0.531
Opacification of anterior capsule	1 (0.7%)	0 (0%)	0.951/0.329

**Table 3:** Comparison of VRQOL score and weighted average LogMAR BCVA in patients undergoing either unilateral or bilateral cataract extraction, ^*^ *P* < 0.05.

	Unilateral cataract extraction patients	Bilateral cataract extraction patients	*P* value (Mann-Whitney test)
Preoperative VRQOL score (SD)	77.02 (30.08)	71.85 (25.24)	0.175
3-month postoperative VRQOL score (SD)	100.32 (20.95)	102.55 (17.65)	0.731
The improvement of VRQOL score (SD)	23.26 (17.89)	30.89 (25.78)	0.112
Preoperative weighted average LogMAR BCVA (SD)	0.76 (0.62)	0.86 (0.44)	0.137
3-month postoperative weighted average LogMAR BCVA (SD)	0.36 (0.36)	0.26 (0. 24)	0.242
The improvement of weighted average LogMAR BCVA (SD)	0.40 (0.37)	0.59 (0.44)	0.024^*^

**Table 4:** Effects of systemic comorbidities, ocular comorbidities, surgical complications, and gender on the improvement of VRQOL score at the end of follow-up period (^*^ *P* < 0.05, ^**^ *0.05* < *P* < 0.10).

	Unilateral cataract extraction patients		Bilateral cataract extraction patients		All cataract extraction patients	
	Mean improvement in score (SD)	*P* value	Mean improvement in score (SD)	*P* value	Mean improvement in score (SD)	*P* value
With systemic comorbidities	25.00 (17.89)	0.671	47.64 (30.58)	0.158	35.83 (26.80)	0.149
Without systemic comorbidities	24.05 (18.89)		26.89 (28.18)		25.40 (23.49)	
With ocular comorbidities	21. 60 (19.31)	0.550	25.94 (35.07)	0.094^**^	24.33 (29.84)	0.135
Without ocular comorbidities	25.61 (18.07)		45.69 (18.45)		32.86 (20.44)	
With surgical complication	7.60 (10.60)	0.019^*^	11.00 (23.45)	0.094^**^	9.11 (16.30)	0.006^*^
Without surgical complication	27.39 (17.78)		38.12 (29.96)		32.56 (24.78)	
Female	25.12 (16.87)	0.763	30.93 (32.39)	0.412	27.84 (25.09)	0.655
Male	23.63 (20.13)		38.07 (28.75)		30.61 (25.34)	

**Table 5:** Multiple impact factors that influence the improvement in VRQOL score at the end of the follow-up period (3 months after cataract surgery) (^*^ *P* < 0.05, ^**^ *0.05* < *P* < 0.10).

	Unilateral cataract surgery patients	Bilateral cataract surgery patients	All cataract surgery patients
Preoperative VRQOL score	−0.367/0.030^*^	−0.823/0.000^*^	−0.679/0.000^*^
Preoperative weighted average LogMAR BCVA	−0.085/0.802	0.055/0.730	−0.554/0.001^*^
Weighted average LogMAR BCVA change	0.461/0.008^*^	0.278/0.053^**^	0.731/0.000^*^
Age	0.088/0.559	0.172/0.176	0.113/0.193
Educational time	0.196/0.116	−0.109/0.323	0.019/0.806
Unilateral or bilateral surgery	/	/	1.220/0.062^**^
With or without surgical complication	−2.015/0.098^**^	−1.802/0.237	−2.190/0.015^*^
With or without systemic comorbidities	−0.045/0.955	1.902/0.133	0.664/0.302
With or without ocular comorbidities	−1.049/0.211	−2.615/0.041^*^	−1.550/0.017^*^
